# Novel mutations in *BMP1* result in a patient with autosomal recessive osteogenesis imperfecta

**DOI:** 10.1002/mgg3.1676

**Published:** 2021-04-05

**Authors:** Lei Xi, Shanshan Lv, Hao Zhang, Zhen‐Lin Zhang

**Affiliations:** ^1^ Shanghai Clinical Research Center of Bone Disease Department of Osteoporosis and Bone Disease Shanghai Jiao Tong University Affiliated Sixth People's Hospital Shanghai China

**Keywords:** bisphosphonates, *BMP1*, novel mutation, osteogenesis imperfecta

## Abstract

**Background:**

Osteogenesis imperfecta (OI) is a rare heritable bone disorder that is characterised by increased bone fragility and recurrent fractures. To date, only 19 OI patients have been reported, as caused by BMP1 gene mutations, worldwide. Here, we report a patient with a BMP1 gene mutation to explore the relationship between genotype and phenotype, and the patient was followed up for 4 years.

**Methods:**

Detailed clinical features were collected, and BMP1 mutational analysis was performed by next‐generation sequencing and Sanger sequencing.

**Results:**

The patient had recurrent fractures, low bone mass, bone deformities and growth retardation but did not have hearing loss or dentinogenesis imperfecta. Next‐generation sequencing and Sanger sequencing revealed a heterozygous novel missense variant (c.362C>T in exon 3, p.Ala121Val) and a heterozygous novel deletion mutation (c.1252delA in exon 10, p.Ser418AlafsX22). The parents of the proband were heterozygous carriers of these mutations. The patient received regular weekly treatment of 70 mg oral alendronate for 3 years, and her BMD Z‐score for the femur significantly increased from −1.3 to 0.9 at L1‐4 and from −1.7 to −0.1. She had no fracture during 4 years of follow‐up.

**Conclusion:**

We discovered two heterozygous novel mutations in an OI patient with BMP1 gene mutations, expanding the spectrum of gene mutations in OI.

## Graphical abstract

Osteogenesis imperfecta (OI) is a rare heritable bone disorder that is characterized by increased bone fragility and recurrent fractures. Up to now, only 19 OI patients have been reported, caused by BMP1 gene mutation around the world. Here, we reported a patient with BMP1 gene mutation, trying to explore the relationship between genotype and phenotype, and the patient was followed up for 4 years.

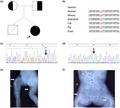

## 1 INTRODUCTION

Osteogenesis imperfecta (OI) is a rare heritable bone disorder that is characterised by increased bone fragility and recurrent fractures, which are caused by defects in the metabolic pathway that is compromised, as follows: collagen biosynthesis; structure, processing and cross‐linking; ossification and mineralisation; posttranslational modification and folding osteoblast differentiation (Forlino, & Marini, 2016). Nearly 90% of cases of OI are caused by mutations of the *COL1A1* (MIM120150) and *COL1A2* (MIM 120160) genes, which encode the α1 and α2 chains of type I collagen. OI individuals are divided into four types according to clinical characteristics and disease severity: dentinogenesis imperfecta (DI), blue sclera, hearing loss and fractures (Sillence et al., 1979). With the emergence of new molecular diagnosis technology, approximately 20 causative genes of OI have been identified, such as *BMP1* (MIM 112264), *IFITM5* (MIM 614757), *P4HB* (MIM 176790), *SERPINF1* (MIM 172860), *CRTAP* (MIM 605497), *SP7* (MIM 606633), *LEPRE1* (MIM 610339), *PPIB* (MIM 123841), *SERPINH1* (MIM 600943), *FKBP10* (MIM 607063), *PLOD2* (MIM 601865), *PLS3* (MIM 300131), *WNT1* (MIM 164820), *SP7* (MIM 606633), *TMEM38B* (MIM 611236), *CREB3L1* (MIM 616215), *SPARC* (MIM 182120), *SEC24D* (MIM 607186), *MESD* (MIM 607783) and *FAM46A* (MIM 611357) (Doyard et al., 2018; Marini et al., 2017; Moosa et al., 2019; Tournis & Dede, 2018).

Bone morphogenetic protein‐1 (*BMP1*)/Tolloid (TLD) is the prototype of the metalloproteinase family, which is involved in the embryonic development of different species. The members of this family contain an astaxanthin‐like protease domain and different numbers of complement subcomponents C1r/C1 s protein–protein interaction domains and epidermal growth factor motifs (Bond & Beynon, 1995). The homozygous 747C‐G transversion in exon 6 of the *BMP1* gene in 2 Egyptian children was first identified in a consanguineous family with severe OI and a large umbilical hernia (Martínez‐Glez et al., 2012).

To the best of our knowledge, only a few reports of OI patients with *BMP1* mutations have previously been reported (Asharani et al., 2012; Cho et al., 2015; Fahiminiya et al., 2015; Martínez‐Glez et al., 2012; Pollitt et al., 2016; Sangsin et al., 2017; Syx et al., 2015; Valencia et al., 2014; Xu et al., 2019). In this study, we detected pathogenic mutations and investigated the phenotypes of a Chinese family with OI caused by compound heterozygous mutations in *BMP1*. In addition, we observed the efficacy of bisphosphonates for the treatment of OI.

## 2 PATIENTS AND METHODS

### 2.1 Subjects

An 8‐year‐old Chinese girl from a non‐consanguineous family and 250 healthy control donors were included in this study. All subjects were Han Chinese. The study was approved by the Ethics Committee of the Shanghai Jiao Tong University Affiliated Sixth People's Hospital, and informed consent was obtained from the parents of the patient and volunteers before blood sampling and DNA analysis.

### 2.2 Ethical Compliance

Our study was approved by the Ethics Committee of Shanghai Jiao Tong University Affiliated Sixth People's Hospital.

### 2.3 Bone densitometry

The bone mineral density (BMD; g/cm2) of the lumbar spine (L1‐L4) was measured by dual‐energy X‐ray absorptiometry (DXA) (Lunar), as conducted by a trained specialist. The coefficient of variability value of the DXA measurements at L1‐4 was 1.39% (Maynard et al., 1998). The BMD results were converted to age‐ and sex‐specific Z‐score‐matched populations (Tan et al., 2007; Xu et al., 2013).

### 2.4 Laboratory tests

The serum level of alkaline phosphatase (ALP) was measured using automated analysers. Other levels were measured using the following kits (all from Roche Diagnostics): a β‐CrossLaps kit for β‐CrossLaps of type I collagen containing cross‐linked C‐telopeptide (β‐CTX) and an osteocalcin kit for osteocalcin (OC). All serum biochemical parameters were measured in the central clinical laboratory of Shanghai Jiao Tong University Affiliated Sixth People's Hospital.

### 2.5 Genetic analysis by next‐generation sequencing

DNA samples of the proband and her parents were extracted from peripheral leukocytes bu conventional methods. A targeted next‐generation sequencing (NGS) panel based on nosology and the classification of genetic skeletal disorders, 2019 revision (Mortier et al., 2019), was designed to capture all sequences of more than 400 genes involved in skeletal disorders, including OI‐related genes (*COL1A1*, *COL1A2*, *IFITM5*, *SERPINF1*, *CRTAP*, *P3H1*, *PPIB*, *SERPINH1*, *FKBP10*, *SP7*, *BMP1*, *TMEM38B*, *WNT1*, *FAM46A*, *CREB3L1*, *SPARC*, *PLOD2*, *PLS3*, *P4HB* and *SEC24D*). NGS was performed using the Illumina Genome Analyser II platform according to the manufacturer's protocol (Illumina, Inc.). To detect variation with high sensitivity and precision, the coverage target of the sample was set at the minimum average depth of 150×.

### 2.6 Sanger sequencing

Sanger sequencing and polymerase chain reaction (PCR) were used to confirm the possible mutations in *BMP1* identified by NGS. Web‐based Primer 3 software (http://bioinfo.ut.ee/primer3‐0.4.0/) was used to design the primers for PCR amplification based on the genomic sequence (NG_029659.1), as follows: forward 5'‐CTGTGCAGGCCGCTTCTG‐3’ and reverse 5'‐TCCCTTGGGAACCCACAATC‐3’; forward 5'‐TTCTACCCCCAGCTGCCAGAGC‐3’ and reverse 5'‐ACACAGGTGTGCTTCTCCCAGTGC‐3'. The patients’ sequences were compared with the Ensembl reference gene sequence ENSG00000168487 (*BMP1*). Direct sequencing was performed using BigDye Terminator Cycle Sequencing Ready Reaction Kit, v. 3.1 (Applied Biosystems, California, USA) and an ABI Prism 3130 automated sequencer. The sequencing results were compared with the OI variant database (https://oi.gene.le.ac.uk/), and variants were defined as novel if not present in the database.

## 3 RESULTS

### 3.1 Clinical features

**FIGURE 1:**
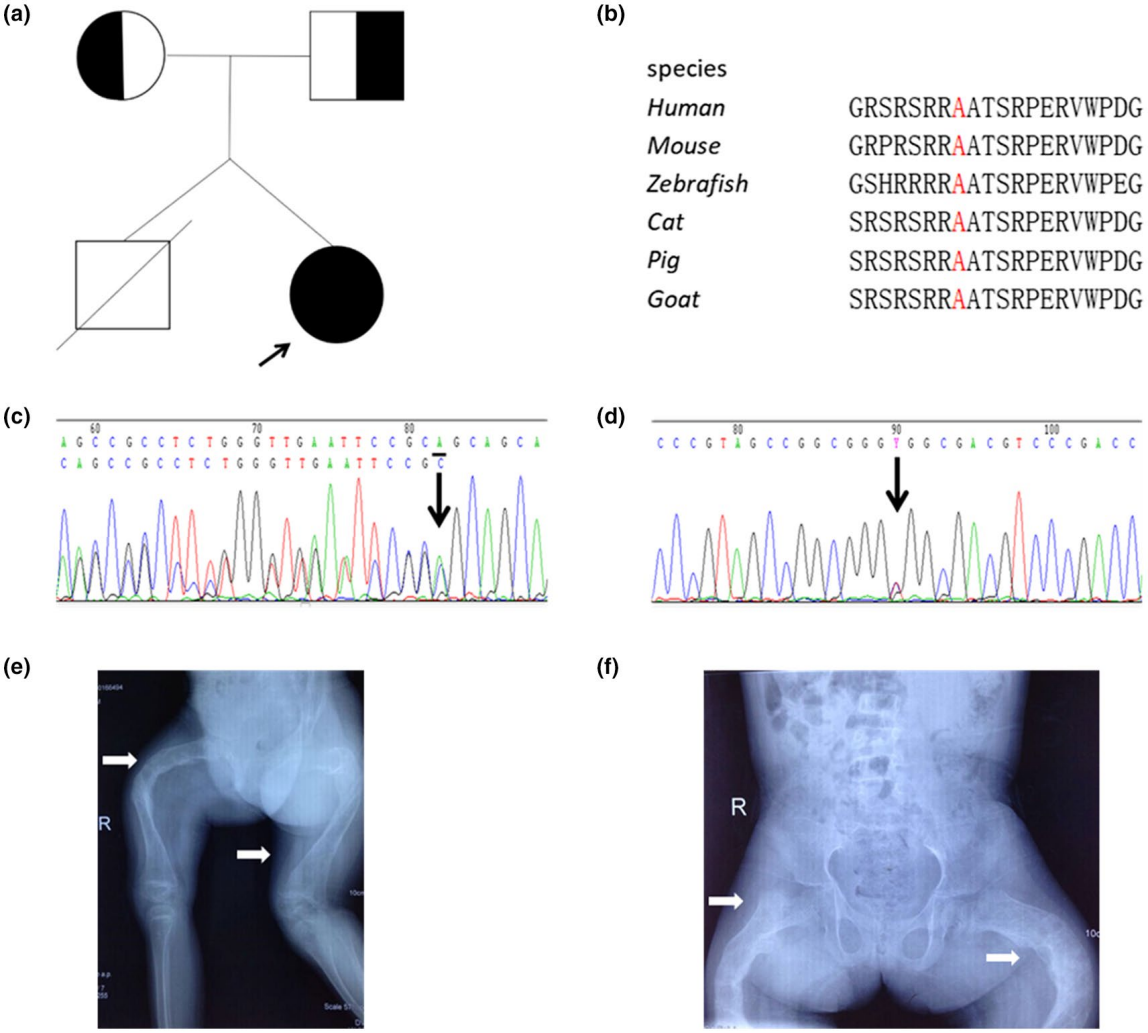
Molecular findings and radiological phenotypes of the patient with OI. (a) The pedigree of the family in this study. The proband is designated with an arrow. (b) A121 is highly conserved among different species. (c) In the proband and her mother, the novel mutation c.1252delA in exon 10 of BMP1 was identified. (d) In the proband and her father, the novel mutation c.362C>T in in exon 3 of BMP1 was identified. (e, f) Radiographs revealed thin cortices and bilateral femur deformities due to repeated fractures

The proband was an 8‐year‐old female, the only surviving daughter of non‐consanguineous parents (Figure 1a). Her twin brother died 10 months after birth (due to infection), without scleral blue, fracture, etc. The proband was born full‐term with a normal delivery, and her birth weight and length were within normal limits. Her height and weight at the time of the study were 110.3 cm (Z score, −3.6) and 20.3 kg (Z score, −1.6), respectively. She experienced her first fracture in the left humerus at the age of 18 months, and she experienced more than 10 additional fractures, including both femurs, the left tibiofibula and the left humerus, between the ages of 1.5 and 8 years. She had blue sclera but did not exhibit DI or hearing loss. Radiographs revealed thin cortices and bilateral femur deformities due to repeated fractures (Figure 1e,f). She had a lower lumbar spine bone mass than healthy controls of the same age. All biological test results were within normal ranges, and her parents did not show any symptoms of OI.

### 3.2 Mutations in *BMP1*

A novel heterozygous missense mutation in exon 3 of *BMP1* (c.362C>T) was identified in the proband and her father; a novel heterozygous deletion mutation in exon 10 of *BMP1* (c.1252delA) was identified in the proband and her mother (Figure 1c,d). Prediction using Mutation Taster software indicated these mutations to be damaging. Moreover, the affected amino acids are highly conserved among different species (Figure 1b), which indicated that the function of these residues has remained important throughout evolution. Detailed information about both variants were given in Table 1.

**TABLE 1:** Detailed information about both variants

	Positon 1	Positon 2
Gene (transcript)	*BMP1*(NM_001199.4)	*BMP1*(NM_001199.4)
Inheritance	Autosomal recessive	Autosomal recessive
Location	EXON3	EXON10
Nucleotide	c.362C>T	c.1252delA
Amino acid	p.Ala121Val	p.Ser418AlafsX22
Zygosity	Heterozygous	Heterozygous
ACMG category	PS2+PM2+PP2+PP3+PP4	PS2+PM2+PM4+PP3+PP4
Classification	Likely pathogenic	Pathogenic

The *BMP1* mutation identified in our patient was absent from 250 unrelated control subjects, and did not match polymorphisms in any public database. No mutation was identified in other candidate genes of OI in this study.

### 3.3 Efficacy of alendronate treatment

**FIGURE 2:**
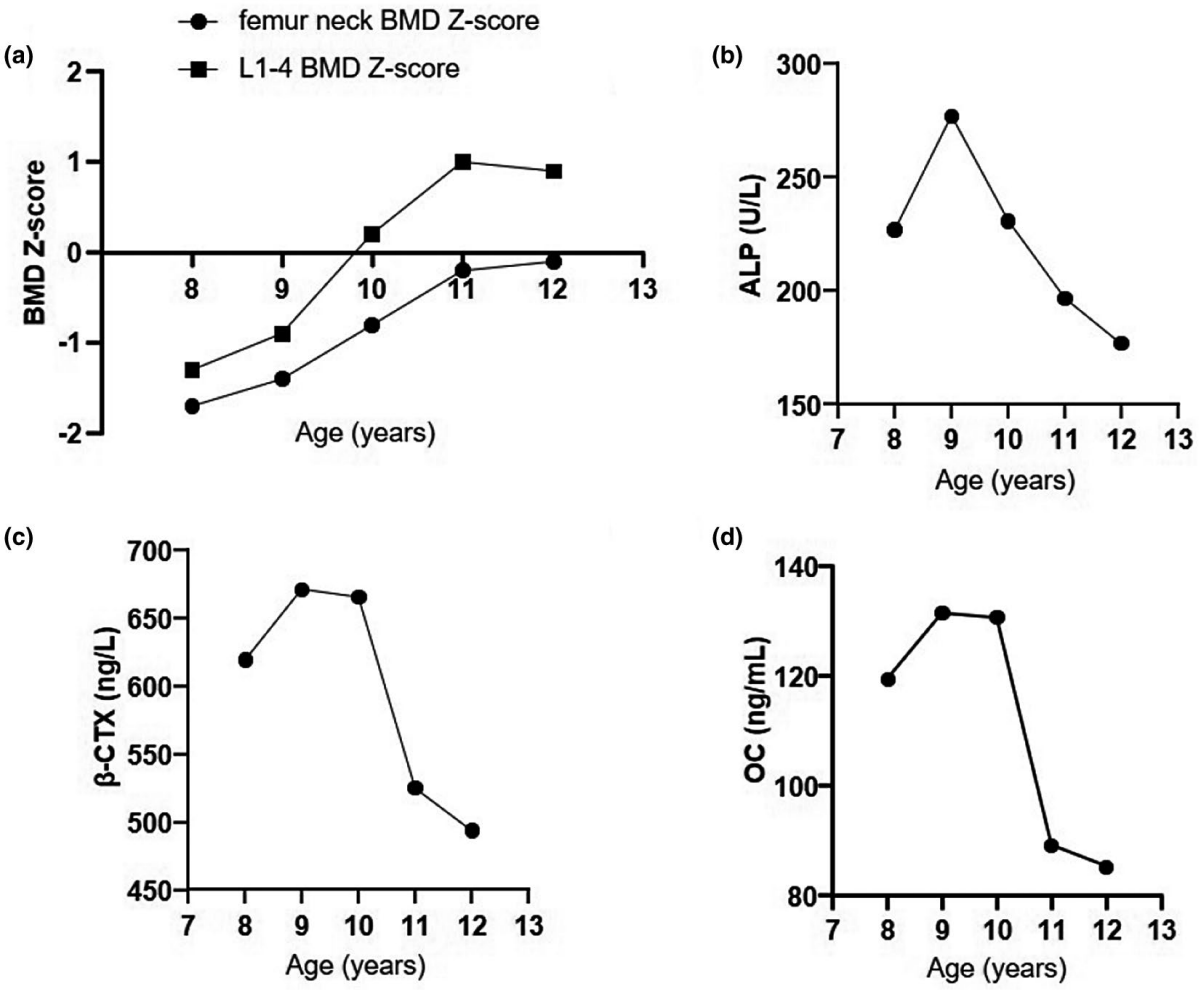
(a–d) Changes in bone turnover biomarkers and BMD Z‐score of the proband after treatment with oral alendronate

As shown in Figure 2, the proband received regular weekly treatment of 70 mg oral alendronate (Fosamax, Merck Sharp & Dohme) from the ages of 8 to 11 years and then entered a drug holiday for 1 year. Her lumbar BMD Z‐score of 1–4 increased significantly from −1.3 to 0.9. Serum levels of β‐CTX, OC and ALP were significantly decreased by 15.2%, 25.4% and 13.2% after 36 months of alendronate and remained at a low level during the drug holiday. No fractures occurred during treatment; her height increased from 110.3 cm (Z‐score −3.6) at 8 years old to 130.0 cm (Z‐score −2.7) at 11 years old, and her weight increased from 20.3 kg to 26.2 kg. Moreover, no obvious adverse events occurred during treatment.

## 4 DISCUSSION

To date, 19 OI patients worldwide have been reported to carry *BMP1* gene mutations (Asharani et al., 2012; Cho et al., 2015; Fahiminiya et al., 2015; Martínez‐Glez et al., 2012; Pollitt et al., 2016; Sangsin et al., 2017; Syx et al., 2015; Valencia et al., 2014; Xu et al., 2019). We identified a Chinese OI patient who had her first fracture at the age of 18 months. She was found to be compound heterozygous for *BMP1* c.362C>T (p.Ala121Val) and c.1252delA (p.Ser418AlafsX22) mutations in the *BMP1* gene and her parents were heterozygous carriers for these mutations. Neither of these mutations has ever been reported. Treatment with alendronate can effectively increase BMD and reduce the number of fractures, but its long‐term effect remains to be determined.

*BMP1*, mammalian TLD (mTLD), and mammalian tolloid‐like 1 and 2 (mTLL 1 and 2) are involved in procollagen I C‐terminal propeptide (PICP) processing. The *BMP1* gene encodes the two proteins BMP1 (730 amino acids) and mTLD, which exhibit the highest efficiency in processing PICP and are mainly expressed in mineralised and soft connective tissues. Some scholars have compared the enzymatic activities and expression domains of four known mammalian *BMP1*/TLD‐like proteases and found distinct differences in the ability to process fibrillar collagen precursors and to cleave Chordin, the vertebrate orthologue of short gastrulation (Scott et al., 1999). As previously demonstrated for BMP1 and TLD, TLL1 specifically processes PICP at the physiologically relevant site, whereas TLL2 lacks this activity. BMP1 and TLL1 cleave chordin at sites similar to those for procollagen C‐propeptide and counteract the dorsalising effects of Chordin upon overexpression in Xenopus embryos. The proteins TLD and TLL2 do not cleave Chordin, which suggests that BMP1 is the major Chordin antagonist in early mammalian embryogenesis and in pre‐ and postnatal skeletogenesis. In addition, BMP1 and BMP1‐like proteinases process prolactin and growth hormone in vitro and in vivo to produce approximately 17‐kD N‐terminal fragments with antiangiogenic activity (Ge et al., 2007).

Our patient in this study showed the most typical characteristics of OI is repeated fracture. Similar to other types of OI, fractures mainly occur in the long bones of limbs, and due to deformity of her lower limbs and her poor health status, she uses a wheelchair. Considering that she is still a child, long‐term follow‐up is needed to assess whether OI will cause hearing loss. Recently, increasing attention has been given to the extraskeletal manifestations of OI and BMP signalling, acting through Smad1, Smad5, and Smad8, which are fundamental hypertrophic signals in mice (Sartori et al., 2013). Inhibition of BMP signalling causes muscle atrophy in mice, which suggests a critical role for the BMP pathway in adult muscle maintenance, growth and atrophy. We evaluated the muscle strength of the patient (Grgic et al., 2018). Although the muscle strength of her upper limbs did not decrease significantly as that of her lower limbs did. It is necessary to further study whether the decrease in lower limb muscle strength is caused by muscle disuse atrophy or OI. For all 20 OI cases caused by *BMP1* mutations identified to date, no significant genotype‐phenotype association similar to type V OI was found. A total of five patients presented with blue sclerae (25.0%), nine probands had scoliosis (45.0%), and one proband had dentinogenesis imperfecta (5.0%); none had hearing loss. The number of fractures per year ranged from 0.7 to more than 15, and the age of first fracture was before 3 years in all patients; in particular, five patients were born with fractures. Umbilical hernia, triangular face, broad forehead, bruising skin and mild ptosis appeared in some patients (Cho et al., 2015; Martínez‐Glez et al., 2012; Pollitt et al., 2016).

Regarding medical treatment, bisphosphonate is the most widely used medical approach to decrease long‐bone fracture rates (Trejo & Rauch, 2016). Our patient presented decreased BMD at the lumbar spine and femoral neck at her first visit. She received regular weekly treatment of 70 mg oral alendronate for 3 years, and her BMD Z‐score increased significantly from −1.3 to 0.9 at L1‐4 and from −1.7 to −0.1 at the femur; she even did not have a fracture during the medication holiday. Interestingly, nearly half of OI patients with *BMP1* mutations (8/20) show increased or normal BMD. Considering the great heterogeneity of clinical phenotypes in patients with OI, we suggest that further study is needed to assess whether high bone mass is related to *BMP1* gene mutations. During the treatment, there were no obvious adverse reactions. However, considering the possibility of adverse reactions such as atypical femoral fractures and the reduction of the number of fractures, we decided to discontinue the drug temporarily and follow‐up. Denosumab (a monoclonal antibody directed against Receptor Activator of Nuclear Factor Kappa B ligand), Teriparatide (the 1–34 fragment of parathyroid hormone) and other drugs also have a certain effect. A small sample of adults with OI were treated with 20 μg teriparatide, and after 18 months of treatment, the BMD of the lumbar spine and femoral neck increased significantly. However, the impact of teriparatide on the fracture rate still needs to be evaluated by a large‐sample study (Orwoll et al., 2014). Treatment with denosumab in children with OI increases BMD, with remodelling of the vertebral body and a reduced fracture rate, but the duration of action of denosumab is short and varies among patients (Hoyer‐Kuhn et al., 2014; Trejo et al., 2018).

In summary, by using next‐generation sequencing, we identified two novel mutations associated with rare types of OI. Although our study expands the spectrum of *BMP1* gene mutations, there were still some limitations, as we did not perform functional analysis of the mutations. Nevertheless, we herein share our experience in treating a rare type of OI.

## CONFLICT OF INTEREST

The authors declare that there are no competing interests associated with the manuscript.

## AUTHOR CONTRIBUTIONS

Lei Xi and Zhen‐Lin Zhang contributed to the conception and design of the study. Lei Xi, Shanshan Lv and Hao Zhang were in charge of the statistical analysis, and Lei Xi drafted the manuscript. Lei Xi, Shanshan Lv and Zhen‐Lin Zhang commented on and revised the draft, and all authors have read and approved the final version of this manuscript.

## Data Availability

The data that support the findings of this study are available from the corresponding author upon reasonable request.
